# Resource-efficient internally controlled in-house real-time PCR detection of SARS-CoV-2

**DOI:** 10.1186/s12985-021-01559-3

**Published:** 2021-06-02

**Authors:** Janine Michel, Markus Neumann, Eva Krause, Thomas Rinner, Therese Muzeniek, Marica Grossegesse, Georg Hille, Franziska Schwarz, Andreas Puyskens, Sophie Förster, Barbara Biere, Daniel Bourquain, Cristina Domingo, Annika Brinkmann, Lars Schaade, Livia Schrick, Andreas Nitsche

**Affiliations:** 1 Centre for Biological Threats and Special Pathogens, Highly Pathogenic Viruses (ZBS 1), Robert Koch Institute, Seestraße 10, 13353 Berlin, Germany; 2 Influenza and Other Respiratory Viruses (FG 17), Robert Koch Institute, Seestraße 10, 13353 Berlin, Germany

**Keywords:** SARS-CoV-2, COVID-19, Diagnostics, Real-time PCR, Internal control

## Abstract

**Background:**

The reliable detection of SARS-CoV-2 has become one of the most important contributions to COVID-19 crisis management. With the publication of the first sequences of SARS-CoV-2, several diagnostic PCR assays have been developed and published. In addition to in-house assays the market was flooded with numerous commercially available ready-to-use PCR kits, with both approaches showing alarming shortages in reagent supply.

**Aim:**

Here we present a resource-efficient in-house protocol for the PCR detection of SARS-CoV-2 RNA in patient specimens (RKI/ZBS1 SARS-CoV-2 protocol).

**Methods:**

Two duplex one-step real-time RT-PCR assays are run simultaneously and provide information on two different SARS-CoV-2 genomic regions. Each one is duplexed with a control that either indicates potential PCR inhibition or proves the successful extraction of nucleic acid from the clinical specimen.

**Results:**

Limit of RNA detection for both SARS-CoV-2 assays is below 10 genomes per reaction. The protocol enables testing specimens in duplicate across the two different SARS-CoV-2 PCR assays, saving reagents by increasing testing capacity. The protocol can be run on various PCR cyclers with several PCR master mix kits.

**Conclusion:**

The presented RKI/ZBS1 SARS-CoV-2 protocol represents a cost-effective alternative in times of shortages when commercially available ready-to-use kits may not be available or affordable.

**Supplementary Information:**

The online version contains supplementary material available at 10.1186/s12985-021-01559-3.

## Background

The new coronavirus SARS-CoV-2 causing the disease COVID-19 was first described in China in the Hubei region at the end of 2019 and evolved into a pandemic within just a few weeks [1]. As with every new infectious disease, the rapid and reliable identification of the causative agent is crucial to the management of diseased patients. Thanks to the advent of Next-Generation Sequencing techniques the first full genome sequence of SARS-CoV-2 could be published only days after the first official reports of the new virus SARS-CoV-2 [2]. Besides several other aspects, the genomic sequence is fundamental to PCR-based diagnostics, and its knowledge enables the assessment of available PCR assays for closely related coronaviruses regarding their usefulness for SARS-CoV-2 detection and, in addition, allows the design of new, potentially more specific PCR assays [3]. For the diagnostics of several pathogens the real-time PCR has become the method of choice, being quick, reproducible, specific, safe and extremely sensitive with detection limits below 10 genome equivalents of a pathogen per reaction [4]. Therefore, it seems obvious that in the recent SARS-CoV-2 pandemic, real-time PCR diagnostics was urgently needed [5].

Fortunately, as early as in mid-January 2020 a first crucial real-time PCR system was published [6] and enabled several diagnostic PCR laboratories all over the world to identify SARS-CoV-2. Since then, many real-time PCR assays were shared in the diagnostics community or published in appropriate journals, either as in-house assays or as commercially available ready-to-use kits with different levels of validation and certification [7]. Both the extensively validated in-house assays as well as the commercial kits have their advantages and drawbacks [8, 9].

Ready-to-use kits that contain all reagents necessary for real-time PCR present the easiest approach to diagnostics, even for laboratories with little experience in virus diagnostics. These kits are sometimes well validated and can have a CE-label. Some include internal controls that check for successful nucleic acid extraction. For many laboratories, these ready-to-use kits can be a perfect solution. However, since in these kits the sequences of primers and probes are usually not released, the user is dependent on the manufacturer regarding the assay's validity for new virus variants. Moreover, some kits are validated for specific real-time PCR instruments, restricting their use, which can be very costly and may become an issue for high throughput analyses. And most important, in the course of the COVID-19 pandemic, several manufacturers had to cope with reagent shortages, leading in some cases to a halt in kit production.

On the other hand, in-house assays, that in principle require a higher expertise to be run routinely in high-throughput, seem to be less susceptible to the problems mentioned above. In most regions of the world, various suppliers can provide primers and probes for real-time PCR. A variety of basic master mixes for PCR and one-step RT-PCR can be obtained from an accordingly huge number of manufacturers. Knowledge of the primer and probe sequence provides some degree of flexibility in adjusting the sequences to newly occurring variants. And when considering the costs for the required individual reagents, in-house assays are generally less costly than ready-to-use kits. However, the ongoing pandemic has been teaching us that oligo suppliers may also suffer from contamination problems and even established manufacturers can run out of reagents, which poses a huge problem to diagnostic laboratories. Finally, the decision between ready-to-use kits and in-house reagents has to fit the individual circumstances.

Here we present the RKI/ZBS1 SARS-CoV-2 protocol that was developed to identify SARS-CoV-2 RNA reliably by targeting two different genomic regions (E-Gene and orf1ab). Both were duplexed with newly designed PCR assays controlling for inhibition (KoMa) as well as successful nucleic acid extraction from respiratory specimens commonly taken for COVID-19 diagnostics (c-myc). While one of the SARS-CoV-2-specific PCRs has already been published [6], the second PCR was newly designed to be highly specific for SARS-CoV-2. The validation of this protocol is presented and the experience with the first 3600 analyzed specimens is used to describe and discuss pitfalls in the interpretation of results.

## Material and methods

### PCR design

The E-Gene assay was taken from [6]. The orf1ab assay was designed based on 72 available sequences at the time of design. Further sequences of coronaviruses, in particular SARS-CoV and the common cold causing coronaviruses OC43, 229E, HKU-1 and NL-63, were included in the alignments to enable a SARS-CoV-2-specific design [10]. KoMa is an artificial sequence that has no significant homology to any sequence in GenBank for which we have established various PCR assays. All assays were designed according to standard rules with the intention to avoid significant interaction in the established duplex PCRs representing the RKI/ZBS1 SARS-CoV-2 protocol and to run at 60 °C annealing temperature. Tables 1 and 2 show the primer and probe sequences used.

**Table 1:** Primers and probes for PCR 1: E-Gene/KoMa

Name	Sequence	O^a^	Position^b^	Tm^c^
E_Sarbeco_F1*	ACAggTACgTTAATAgTTAATAgCgT	S	26,141	53.9
E_Sarbeco_R2*	ATATTgCAgCAgTACgCACACA	A	26,232	57.9
E_Sarbeco_P1*	FAM-ACACTAgCCATCCTTACTgCgCTTCg-BHQ1	S	26,204	65.0
KoMa F	ggTgATgCCgCATTATTACTAgg	S	n/a^d^	57.8
KoMa R	ggTATTAgCAgTCgCAggCTT	A	n/a^d^	57.8
KoMa TM	TexRed-TTCTTgCTTgAggATCTgTCgTggATCg-BBQ	S	n/a^d^	67.7

^a^Orientation

^b^Position in GenBank entry (E_Sarbeco: Acc# NC_004718)

^c^Thermodynamic melting temperature

^d^Artificial sequence

^*^Corman et al.

**Table 2:** Primers and probes for PCR 2: orf1ab/c-myc

Name	Sequence	O^a^	Position^b^	Tm^c^
orf1ab S	CTCTggAACACTTTTACAAgACTTC	S	19,608	54.5
orf1ab A	ACCATCAACTTTTgTgTAAACAgTg	A	19,754	56.3
orf1ab TMGB	FAM-ACAgggTgAAgTACCA-MGB	S	19,688	66.0
c-myc F	TAgTggAAAACCAgCAgCCT	S	380	57.0
c-myc R	TCgTCgCAgTAgAAATACgg	A	488	56.0
c-myc TM	Cy5-TATgACCTCgACTACgACTCggTgC-BBQ	S	442	63.5

^a^Orientation

^b^Position in GenBank entry (orf 1ab: Acc# MN997409.1; c-myc NM_002467.6, GeneID: 4609)

^c^Thermodynamic melting temperature

^*^Corman et al. [6]

### Principle of the assays

The established RKI/ZBS1 SARS-CoV-2 protocol is a one-step real-time RT-PCR assay that consists of two independent PCR reactions. PCR 1 includes the already published E-Gene assay with a FAM-labeled probe, but is adapted to 60 °C annealing temperature. The E-Gene assay is duplexed with an assay to detect the artificial sequence KoMa which is added as VLP to the clinical specimen prior to nucleic acid extraction and ideally results in a CT value of approximately 32. A significant CT value shift for KoMa indicates PCR inhibition or inefficient RNA extraction. The KoMa assay uses two primers and a Texas Red-labeled probe.

PCR 2 includes a PCR assay located in the orf1ab gene region with two primers and a FAM-labeled Minor Groove Binding (MGB) probe for SARS-CoV-2 detection. Based on this MGB probe, the orf1ab assay is highly specific for SARS-CoV-2. The orf1ab assay can be duplexed with an assay to detect human nucleic acid, DNA as well as RNA/cDNA, with two primers and a Cy5-labeled probe. In particular for SARS-CoV-2-negative specimens, the proof of nucleic acid in the PCR reaction is unequivocal to exclude failure in sampling.

**Fig. 1:**
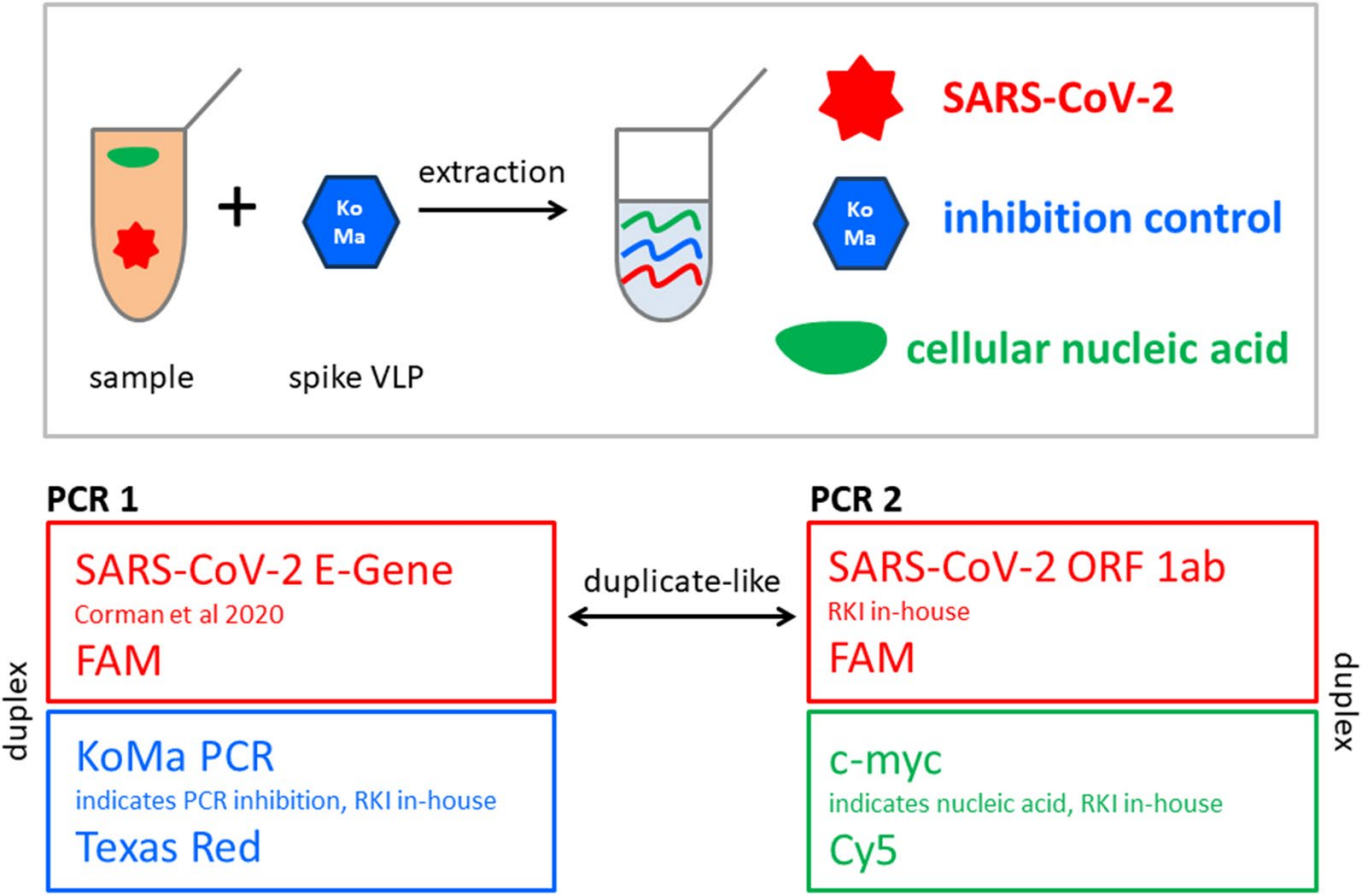
Principle of the RKI/ZBS1 protocol for the real-time PCR detection of SARS-CoV-2. RKI/ZBS1 SARS-CoV-2 protocol

Due to the fact that the detection limit and PCR efficiency of the E-Gene assay and the orf1ab assay are nearly identical, as determined by probit analyses, we recommend running both PCR 1 and PCR 2 as "duplicate-like" assays to save patient material and PCR consumables, increasing the sample throughput at the same time. Additionally, the use of two individual PCRs in two wells of the same PCR plate instead of commonly used duplicates draws attention to pipetting errors or other unexpected discrepancies by keeping a conformational approach. Figure 1 summarizes the principle of the RKI/ZBS1 SARS-CoV-2 protocol.

### In silico validation of the SARS-CoV-2 PCR assays

With the increasing number of genomic sequences deposited in public databases like GISAID, the primers and probes applied for SARS-CoV-2 detection in PCR 1 and PCR 2 were subjected to controls by aligning the oligonucleotide sequences to full genome alignments of all available SARS-CoV-2 genome sequences. Common PCR design rules were applied to assess the validity of the PCR assays [11, 12].

### Reaction conditions

The presented RKI/ZBS1 SARS-CoV-2 protocol has been established, validated and subsequently used in routine diagnostics with the following workflow. RNA extracted by the QIAamp Viral RNA Mini Kit (Qiagen, Hilden, Germany) and PCR performed with the AgPath-ID™ One-Step RT-PCR Reagents kit (Applied Biosystems, Foster City, CA, USA) on a Bio-Rad CFX96 real-time PCR cycler. Cycling conditions were set as follows: 45 °C for 15 min, 95 °C for 10 min followed by 45 cycles of 95 °C for 15 s and 60 °C for 30 s. Total runtime was about 95 min. PCR mix composition is shown in Tables 3 and 4. As positive controls genomic SARS-CoV-2 RNA and genomic SARS-CoV RNA were used for the orf1ab and the E-Gene assay, respectively, and adjusted to CT 28 and 32.

**Table 3:** Master mix PCR reaction 1 E-Gene/KoMa

Reagent	Vol. (µL)
Water	2.25
2× RT-PCR buffer	12.50
nCoV E_Sarbeco F1 (10 µM)	1.00
nCoV E_Sarbeco R2 (10 µM)	1.00
E_Sarbeco P1 FAM (10 µM)	0.50
KoMa F (10 µM)	0.75
KoMa R (10 µM)	0.75
KoMa TM TexRed (10 µM)	0.25
25× RT-PCR Enzyme mix	1.00
Mix vol	20.00
RNA vol	5.00
Total vol	25.00

Volumes used for reaction set up, concentrations of stock solutions are given in brackets

**Table 4:** Master mix PCR reaction 2 orf1ab/c-myc

Reagent	Vol. (µL)
Water	3.9
2× RT-PCR buffer	12.50
orf1ab S (10 µM)	0.75
orf1ab A (10 µM)	0.75
orf1ab TMGB (10 µM)	0.25
c-myc F (10 µM)	0.3
c-myc R (10 µM)	0.3
c-myc TM Cy5 (10 µM)	0.25
25× RT-PCR Enzyme mix	1.00
Mix vol	20.00
Sample vol	5.00
Total vol	25.00

Volumes used for reaction set up, concentrations of stock solutions are given in brackets

To assess the performance of further commercially available one-step RT-PCR master mixes with the RKI/ZBS1 SARS-CoV-2 protocol, 10 clinical specimens with varying viral genome load were analyzed with the AgPath-ID master mix on the Bio-Rad CFX96 cycler and compared to the SuperScript™ III One-Step RT-PCR System with Platinum™ Taq DNA Polymerase (Invitrogen, Carlsbad, CA, USA), the lyophilized 1-step RT-PCR Polymerase Mix (TIB MOLBIOL, Berlin, Germany), the TaqPath™ 1-Step Multiplex Master Mix (No ROX) (Applied Biosystems), two kits from Solis BioDyne (SOLIScript 1-step Multiplex Probe Kit, SOLIScript® 1-step CoV Kit; Solis BioDyne, Tartu, Estonia) and the GoTaq® Probe 1-Step RT-qPCR System (Promega, Walldorf, Germany). All PCR reagents were used according to the manufacturers' instructions, with the exception of 5 µl of template used for all kits for better comparability. When 10 µl of template were recommended, the reaction volume was substituted with water. In addition, five popular real-time PCR cyclers were compared with the same specimen panel by using the standard protocol based on the AgPath-ID kit shown above. These cyclers included: the Bio-Rad CFX96 (CFX96 Touch Real-Time PCR Detection System), the AB7500 (7500 Real-Time PCR System, Applied Biosystems), the AB Quantstudio 5 (QuantStudio 5 Real-Time PCR System, Applied Biosystems), the LC480 (LightCycler® 480 Instrument II, Roche, Mannheim, Germany) and the Rotor-Gene (Rotor-Gene Q 5plex Platform, Qiagen).

### Specificity testing

Specificity of the E-Gene assay has already been shown in the original publication [6]. Specificity of the orf1ab PCR assay in PCR 2 was assessed with several clinical specimens from patients with respiratory infections as well as cell culture supernatant of relevant viruses (for details see results section). These specimens showed CT values between 10 and 22 with virus-specific assays.

### Limit of detection

Probit analyses [13] were performed for the uniplex E-Gene and orf1ab PCR, respectively, in direct comparison to the duplex PCR 1, E-Gene/KoMa, and PCR 2, orf1ab/c-myc. Dilutions of SARS-CoV-2 genomic RNA were quantified by comparison to in vitro RNA prepared in a background of SARS-CoV-2-negative patient swab material to simulate typical amounts of human nucleic acid background, extracted and subjected to the uniplex SARS-CoV-2 assays and the duplex PCR assays PCR 1 and PCR 2. Duplicates were run for clearly detectable virus amounts down to 75 genome copies per reaction, while tenfold replicates were run for concentrations below this.

### KoMa virus-like particle (VLP) production

KoMa VLPs were generated according to standard protocols with minor specific modifications (to be published elsewhere). The protocol including the sequence as well as limited amounts of VLPs containing the KoMa sequences can be provided to interested parties on request. The Qiagen (QIAamp Viral RNA Mini QIAcube Kit) extraction protocol included 5 µL of KoMa VLP suspension added to AVL buffer prior to adding of the specimen. Following extraction, the expected CT value for KoMa is in the range of 32 in PCR 1 (E-Gene/KoMa). FCS spiked with KoMa was extracted to define the actual reference value and to control the VLP quality.

### Clinical specimens

Dry or wet nasopharyngeal and/or oropharyngeal swabs were sent to the Robert Koch Institute for PCR diagnostics. Wet swabs were kept in transport media, dry swabs were transferred to 500 µL of PBS. If two swabs arrived for one person, liquid for extraction was pooled. All swabs were vortexed and spun down prior to extraction of 140 µl of the specimen, by using the QIAamp Viral RNA Mini Kit and QIAcube Connects with the manual lysis protocol. Remaining specimen volume was stored at − 40 to − 80 °C.

## Results

### Specificity and limit of detection

Information regarding the E-Gene assay analytical specificity can be taken from [6]. Analytical specificity testing for the SARS-CoV-2 orf1ab uniplex assay showed no reactivity for SARS-CoV (cell culture n = 1), MERS-CoV (cell culture n = 1), NL63, OC43, 229E (each cell culture n = 1) and patient samples infected with parainfluenza virus 1 (n = 15) and 4 (n = 4), rhinovirus (n = 20), influenzavirus A (H1N1 n = 1, H3N2 n = 20), influenzavirus B (n = 1), adenovirus (various, n = 20), human metapneumovirus (n = 20) and respiratory syncytial virus (n = 17) when tested in high viral loads of CT values between 10 and 22.

In addition, due to the fact that not all new SARS-CoV-2 variants can be tested in practice, the RKI/ZBS1 SARS-CoV-2 protocol was validated in silico twice a week. Of the 76,312 high-coverage genome sequences acquired from the GISAID database (on Oct 15, 2020), primer and probe mismatches could be identified in 2% of the sequences, rarely in essential positions, indicating the assay’s functionality for the vast majority of circulating viruses. However, a regular control for the regionally circulating viruses with primer and probe adaption is strongly recommended, as long as the changes do not conflict with the assay's reaction temperature and specificity.

By probit analysis the limit of detection for the uniplex E-Gene assay under the described standard reaction conditions (AgPath-ID PCR kit, BioRad CFX96) was determined as 6.4 genome copies per reaction in comparison to 9.8 genome copies in the duplex E-Gene/KoMa PCR 1. For the orf1ab assay the probit analysis revealed 6.6 genome copies per reaction for both the uniplex PCR and the duplex orf1ab/c-myc PCR 2. When comparing the results obtained for the orf1ab assay to the results generated by the E-Gene assay, which can be considered as reference, for 424 SARS-CoV-2-positive specimens, the sensitivity of the orf1ab assay was 97.6%, the specificity 99.8%, the positive predictive value 98.8% and the negative predictive value 99.7%.

Additional file 1: Figure S1 shows the correlation of the CT values obtained for 407 specimens positive for the E-Gene PCR with the orf1ab PCR. Shown is the correlation over all specimens (blue line, R^2^ = 0.94), the correlation for specimens with E-Gene PCR CT values below CT = 33 (red line, R^2^ = 0.95) and finally also for specimens with E-Gene PCR CT values above CT = 33 (green line, R^2^ = 0.33).

Moreover, the established RKI/ZBS1 SARS-CoV-2 protocol was compared to the RealStar® SARS-CoV-2 RT-PCR Kit 1.0 (Altona Diagnostics, Hamburg, Germany) and the EURORealTime SARS-CoV-2 (EuroImmun, Lübeck, Germany) with 10 clinical specimens described below. Differences in CT values were < 0.2 when comparing the RKI/ZBS1 SARS-CoV-2 protocol with the commercially available kits, indicating highly similar detection capabilities.

### Experience from routine application of the RKI/ZBS1 SARS-CoV-2 protocol

**Fig. 2:**
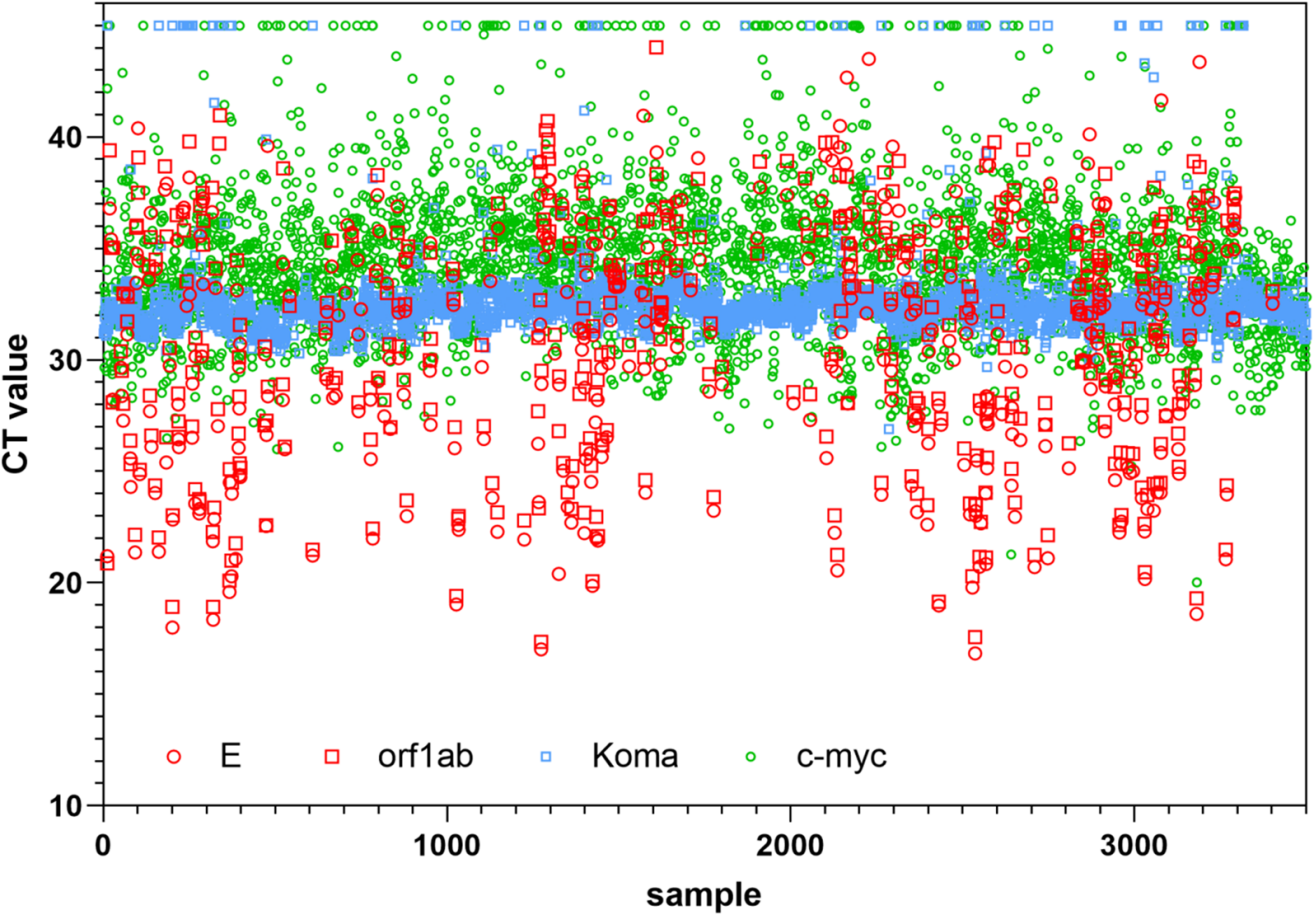
CT value overview for 3600 clinical specimens tested with the RKI/ZBS1 SARS-CoV-2 protocol. CT values for the four PCR assays included in the RKI/ZBS1 SARS-CoV-2 protocol as determined for the first 3600 specimens. Red symbols show the SARS-CoV-2 assays (negatives not shown), blue squares the inhibition control KoMa and green circles the nucleic acid detection by c-myc amplification. The constant detection of the inhibition control KoMa can be seen, and the scattering of the nucleic acid content demonstrated by c-myc as well as the similar CT values for the E-Gene and the orf1ab assay

The presented RKI/ZBS1 SARS-CoV-2 protocol aims at a high sample throughput with resource-friendly reagent consumption. Therefore, we did not run duplicates per reaction (PCR 1: E-Gene/KoMa and PCR 2: orf1ab/c-myc), but instead treated the results of the two different SARS-CoV-2 assays, E-Gene and orf1ab, like a duplicate. Figure 2 displays the results of the first 3600 naso- and oropharyngeal specimens analyzed with the RKI/ZBS1 SARS-CoV-2 protocol. Red icons show the CT values obtained for 424 SARS-CoV-2-positive specimens (11.8%) with a difference of mean CT values of 30.5 [± 5.4] for the PCR 1 and 30.9 [± 5.3] for the PCR 2. Negative samples are displayed as CT 45 since PCR is run for 45 cycles; however, the value of 45 is not used for any calculation.

Furthermore, Fig. 2 shows that the CT values for the nucleic acid control c-myc in PCR 2 are scattered between CT 20 and CT 44, reflecting the varying nucleic acid content of the swab samples. Only 90 out of 3600 specimens (2.5%) were negative for c-myc, with 8/90 being clearly positive for SARS-CoV-2, making the c-myc result obsolete. The remaining 82 c-myc-negative samples can probably be attributed to inefficient swabbing.

**Fig. 3:**
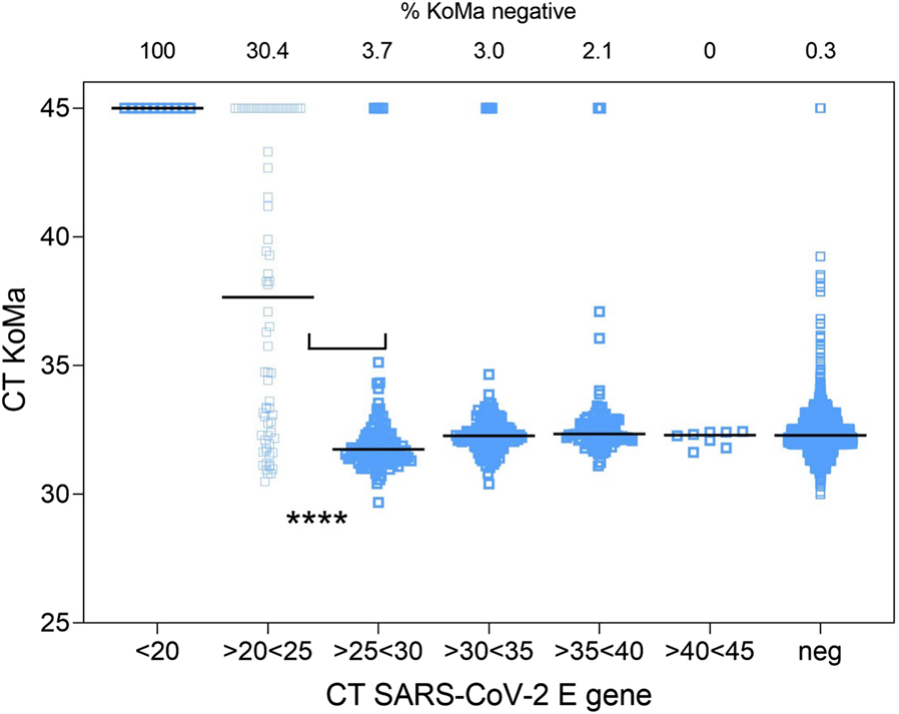
Correlation between the internal KoMa control and SARS-CoV-2 genome load in clinical specimens. For the 424 SARS-CoV-2-positive specimens the CT values were grouped as indicated and plotted against the CT value for the inhibition control KoMa. While in specimens that are highly positive for SARS-CoV-2 KoMa does not give a signal reliably, in specimens with genome loads close to the detection limit as well as in negative samples, KoMa is constantly amplified, showing the efficiency of the nucleic acid extraction and possible inhibitory effects of the sample matrix. Detectability increases significantly with E-Gene CT values higher than 25 (Mann Whitney *p* < 0.0001)

The mean KoMa CT value of the KoMa-positive samples is in the expected range of 32.3 ± 1.4. Figure 3 shows the general principle of the KoMa control. While highly positive SARS-CoV-2 specimens with CT values < 20 and < 25 showed either no or only 70% KoMa positives, respectively, a decreasing SARS-CoV-2 genome load (CT > 25) led to a significantly higher positivity rate for KoMa detection (*p* < 0.0001). Of the SARS-CoV-2-negative specimens, only 0.3% failed to amplify KoMa, pointing to PCR inhibition or extraction failure. This is most important, since in qualitative settings, like the one described above, samples that are clearly positive for SARS-CoV-2 do not need confirmation by the inhibition control. Only samples that are negative or borderline positive for SARS-CoV-2 require proof that the PCR is not inhibited and RNA extraction was efficient.

### Challenges of result interpretation

The huge majority of approximately > 98% of the analyzed specimens gave clear-cut results after the first run, either negative or positive. However, to facilitate result interpretation for ambiguous results, Table 5 summarizes the possible constellations together with the proposed required measure for confirmation. Assuming that the amplification curves are real amplification curves, even appearing late or low, the following scheme was referred to for result interpretation.

**Table 5:** Interpretation of results obtained with the RKI/ZBS1 SARS-CoV-2 protocol

	SARS-CoV-2		Inhibition control	Nucleic acid control	Result	Comments
#	E-Gene	orf1ab	KoMa	c-myc		
1	< 38	< 38	Any	Any	Positive	
2	< 38*	≤ 45*	32 ± 3	Signal	Positive	
3	≤ 45*	< 38*	32 ± 3	Signal	Positive	
4	≥ 38	≥ 38	32 ± 3	Any	Ambiguous	if curves are distinctly positive; if not, analyze follow-up sample
5	Signal	Negative	32 ± 3	Signal		repeat PCR with 10 µl
6	Negative	Signal	32 ± 3	Signal		repeat PCR with 10 µl
7	Negative	Negative	32 ± 3	Negative	Invalid	analyze follow-up sample
8	Negative	Negative	> 35	Any	Invalid	repeat extraction with 1:10 dilution
9	Negative	Negative	32 ± 3	Signal	Negative	

Besides clearly positive and clearly negative results showing the controls as expected, unequivocal results can be obtained in particular with low concentrations of viral RNA. Measures for confirmation of such results are described in the table

^*^If ΔCT value is > 3 repeat PCR with 10 µl of RNA template

If the RKI/ZBS1 SARS-CoV-2 protocol is repeated with 10 µl of template RNA according to the scheme in Table 5, for “real” PCR results (contrasted to PCR artifacts) a shift by approximately one CT value is expected.

### The RKI/ZBS1 SARS-CoV-2 protocol can be used with several PCR reagent kits and real-time PCR cyclers

**Fig. 4:**
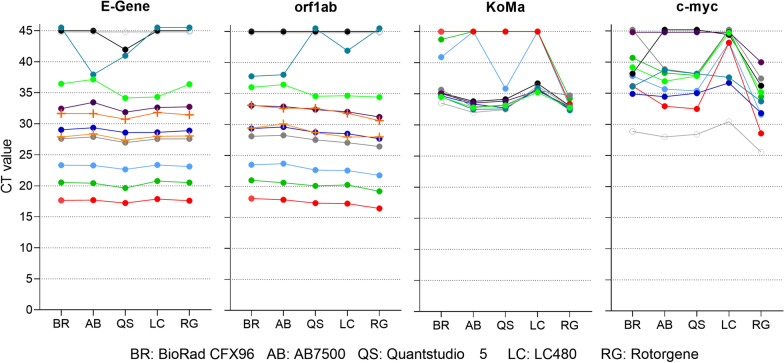
RKI/ZBS1 SARS-CoV-2 protocol performance on different PCR cyclers. To show that the presented protocol can be run on several cyclers, we used 10 clinical samples of different RNA load, negative as well as positive controls and set up one master mix that was distributed to the different cyclers as shown above. Different colors represent 10 different samples used for comparison. Mean values of duplicates are shown, also for SARS-CoV-2-positive controls (crosses); negative controls are not shown

We tested a selection of 10 clinical specimens on five popular real-time PCR cyclers as listed in the Material section. Figure 4 displays the CT values obtained on the different instruments for the E-Gene, the orf1ab, the KoMa and the c-myc assays. Even if a statistically sound conclusion cannot be drawn from these resource-friendly generated data, it can be assumed that for virus loads with CT values below 35 the differences for SARS-CoV-2 detection are marginal between the cyclers. It can be assumed that the Rotor-Gene copes better with the orf1ab assay than with the E-Gene assay, as shown by a detection that was by 1–2 CT values lower for the same specimens. For the LC480 this effect is less distinct. Lower virus loads lead to CT scattering between different instruments as expected for statistical reasons. However, no clear trend towards one or the other cycler can be observed when assessing the virus detection.

Evaluating the control assays KoMa and c-myc, it is peculiar that the Rotor-Gene is able to detect KoMa as well as c-myc in all 10 clinical specimens, independent of the viral genome amount amplified in the duplex PCR reactions. All other instruments show dropouts in the control reactions.

The runtime of the RKI/ZBS1 SARS-CoV-2 protocol for the different instruments was 92 min for the Bio-Rad CFX96, 90 min for the AB7500, 90 min for the AB Quantstudio 5, 95 min for the LC480 and 111 min for the Rotor-Gene.

### PCR master mixes

**Fig. 5:**
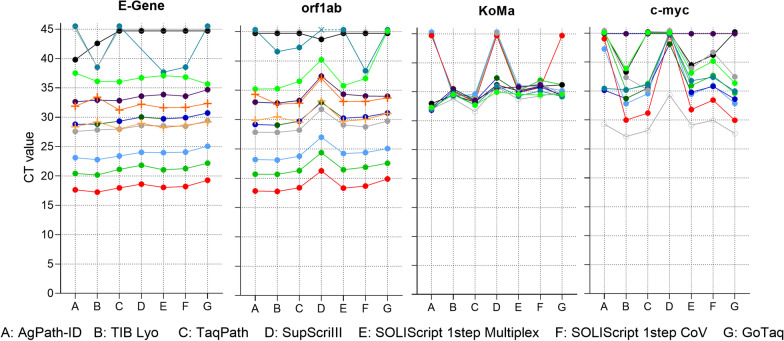
RKI/ZBS1 SARS-CoV-2 protocol performance with different PCR master mixes. In total 7 PCR mixes were compared with the RKI/ZBS1 SARS-CoV-2 protocol on a Bio-Rad CFX96 cycler. 10 clinical specimens of different RNA load and negative as well as positive controls were set up. Means of duplicate CT values are plotted against the respective master mix. Positive controls (crosses) are shown only for the SARS-CoV-2 assays E-Gene and orf1ab; negative controls are not shown

Seven one-step RT-PCR master mixes were compared with the RKI/ZBS1-SARS-CoV-2 protocol. As shown in Fig. 5, up to CT 35 all kits perform comparably, with the exception of the SuperScript™ III One-Step RT-PCR System with Platinum™ Taq DNA Polymerase and the GoTaq® Probe 1-Step RT-qPCR System. The first one shows slightly increased CT values with the E-Gene assay in PCR 1 and clearly increased CT values for the orf1ab assay in PCR 2. The second one gives slightly higher CT values for both SARS-CoV-2 assays. For specimens containing only very low virus amounts, no clear conclusion can be drawn. For one specimen tested negative several times also with various master mixes, a low positive signal of CT 38.5 could be observed with the TIB MOLBIOL reagent. Similar results occurred occasionally with the E-Gene assay in PCR 1 independently of the reaction conditions, but could be clarified by comparison with the orf1ab assay in PCR 2 according to Table 5. Finally, as observed in the cycler comparison, the duplexed control assays KoMa and c-myc seem to be more susceptible to the master mix under the applied standard reaction conditions. The lyophilized 1-step RT-PCR Polymerase Mix (TIB MOLBIOL) and the SOLIScript® 1-step CoV kit can amplify the KoMa control even in specimens highly positive for SARS-CoV-2, even with higher CT values than expected. Similarly, the duplexed c-myc assay seems to be more sensitive, with these reagents being positive in ten out of ten specimens with the SOLIScript® 1-step CoV kit, while the SuperScript™ III One-Step RT-PCR System with Platinum™ Taq DNA Polymerase amplifies c-myc only in two out of ten specimens from the same specimens.

## Discussion

In this study we developed a protocol that can be considered as a robust alternative to available protocols. This RKI/ZBS1 SARS-CoV-2 protocol detects two different SARS-CoV-2 genomic regions in independent reactions. The E-Gene assay has already been published; the orf1ab assay was established and validated in this study. Each of the SARS-CoV-2 assays is combined with a control in a duplex reaction that enables either inhibition control or efficient sampling, respectively. Especially for SARS-CoV-2-negative specimens these controls are crucial for reporting a reliable result. Hence, the inhibition control KoMa was established to give a signal in negative and low positive specimens. In highly positive SARS-CoV-2 specimens KoMa is not amplified, which is acceptable since clearly positive specimens do not require a proof of an unaffected PCR reaction in a qualitative setting. The c-myc assay was selected after testing several known "reference genes", including assays that exclusively amplify RNA and not genomic DNA [14]. Unfortunately, we observed that RNA-specific assays became positive in 50%–70% of the analyzed specimens. Based on the available data, we could not find a correlation to swab types, sampling site or SARS-CoV-2 positivity. In contrast, a considerable proportion of SARS-CoV-2-positive specimens were negative in the RNA control assay to indicate presence of RNA. It was assumed that the tested assays were not sensitive enough to amplify the amounts of cellular mRNA in the investigated specimens that were sometimes extremely low, possibly due to the denaturation of free cellular RNA at room temperature [15]. Therefore, we redesigned the control PCR to amplify RNA as well as DNA. With this assay we succeeded in showing correct sampling for 97.5% of the 3600 specimens described here.

Notably, even if real-time PCR is generally able to provide quantitative results, it is not possible to quantify SARS-CoV-2 from swab specimens. As demonstrated by the varying CT values for c-myc, swabs do not permit a standardization that could be used as reference for quantification, such as the one that blood, urine or other bodily fluids can provide via the volume. Hence, quantitative results should not be expected from PCR diagnostics of swabs.

The comparison of five common real-time PCR cyclers with the standard protocol showed that for SARS-CoV-2 loads of up to CT 35 no significant differences could be observed. They can be dependent on the assay, as the Rotor-Gene was providing CT values that were 1–2 units lower for the orf1ab assay compared to other instruments, but not for the E-Gene assay. However, even if a full statistical evaluation with multiple repetitions may be required to reveal minor variances for virus specimens at low concentrations, it can be hypothesized that, at least after careful adaption of the reaction conditions, all cyclers are suitable for SARS-CoV-2 detection with the primers and probes presented. More striking differences could be observed regarding the ability to amplify the duplexed control reactions KoMa and c-myc. Here, the Rotor-Gene was the only instrument to amplify KoMa as well as c-myc in the respective duplex PCR assays, regardless of the virus concentration in the specimen. Since the control reactions were established with low primer and probe concentrations to reduce the impact on the virus detection, the Rotor-Gene seems to manage best these sub-optimal reaction conditions.

However, in addition to the detection limit, several other factors have to be considered to select the proper real-time PCR cycler, such as availability, costs for the acquisition and the consumables as well as practical questions of workflow automation [16, 17].

For the master mix comparison seven reagents were selected. As reference the routinely used AgPath-ID™ One-Step RT-PCR Reagents kit (Applied Biosystems) was compared to three master mixes that are supposed to be stable at room temperature for one to four weeks, promising good handling even in laboratories not able to provide constant cold chains, from TIB MOLBIOL and Solis BioDyne, as well as commonly used master mixes listed in the material section. For viral loads with CT values below 35 most kits performed comparably well. Interestingly, with the E-Gene assay the SuperScript™ III One-Step RT-PCR System with Platinum™ Taq DNA Polymerase generated similar CT values to most of the other kits, but showed clearly elevated CT values for all other assays, orf1ab, KoMa and c-myc. There is no obvious similarity between these three assays, except that they run with lower primer and probe concentrations than the E-Gene assay. Moreover, even when the GoTaq® Probe 1-Step RT-qPCR System showed slightly elevated CT values for all four assays there was no assay-specific effect, and all SARS-CoV-2-positive specimens were identified correctly. As shown in Fig. 5, the control reactions KoMa and c-myc were less often amplified with the SuperScript™ III One-Step RT-PCR System with Platinum™ Taq DNA Polymerase and showed higher CT values than other master mixes did. It can be speculated that these master mixes require higher primer and probe concentrations or are not composed to run duplex PCRs. However, no total failure was observed for any of the tested kits as had been surprisingly observed previously in other settings [18]. The yield in amplifying the controls was best with the TIB MOLBIOL and the SOLIScript® 1-step CoV Kit.

It is important to note that beside the system-inherent variability of real-time PCR, in particular in the low concentration range of a target, individual effects like the definition of the fluorescence signal threshold or the probe quality may have an influence on the results.

Summing up, we have established a real-time one-step RT-PCR-based protocol that can be used to detect SARS-CoV-2 RNA in clinical specimens with low detection limit. By duplexing two independent SARS-CoV-2 detection assays with an inhibition control and a nucleic acid control, the protocol is highly reliable regarding false negative samples due to sequence variation in the virus genome, poor sampling, extraction or PCR inhibition. The protocol saves reagents by running a duplicate-like approach and can be transferred to various real-time PCR instruments and PCR master mixes. Because of its flexibility, this protocol presents a helpful alternative to other solutions in times of reagent shortage.

Finally, the reliability of the RKI/ZBS1 SARS-CoV-2 protocol could be proven by the successful participation in four external quality assessment studies (three times INSTAND, one time ECDC); results are going to be published by the ring trial providers.

## Conclusions

Although a variety of real-time PCR assays for the detection of SARS-CoV-2 has been published to date, the presented RKI/ZBS1 SARS-CoV-2 protocol shows some advantages. First, it detects two independent SARS-CoV-2 genomic regions that can be used for confirmation instead of using duplicates of the same target. Second, it utilizes two controls proving the successful sampling by indicating cellular nucleic acid, which is particularly important for SARS-CoV-2-negative specimens, as well as inhibition of the PCR process. Since all four assays can be run in two duplex PCR reactions, the presented approach saves time and resources, which becomes more and more important in times of PCR reagent and consumable shortages.

## Supplementary Information

**Additional file 1: Figure S1.** Correlation of the CT values obtained for 407 specimens positive for the E-Gene PCR with the orf1ab PCR. Shown is the correlation over all specimens (blue line, R^2^ = 0.94), for specimens with E-Gene PCR CT values below CT = 33 (red line, R^2^ = 0.95) and for specimens with E-Gene PCR CT values above CT = 33 (green line, R^2^ = 0.33).

## Data Availability

KoMa VLPs can be obtained on request.

## Abbreviations

CT: Threshold cycle
FCS: Fetal calf serum
MGB: Minor Groove Binder
PCR: Polymerase chain reaction
RKI: Robert Koch Institute
RT-PCR: Reverse-transcription polymerase chain reaction
VLP: Virus-like particle
ZBS 1: Centre for Biological Threats and Special Pathogens, Highly Pathogenic Viruses
