# Supplementary figures and images for: Resource-efficient internally controlled in-house real-time PCR detection of SARS-CoV-2
Source: Virol J. 2021 Jun 2;18:110. doi: 10.1186/s12985-021-01559-3 (PMC8170437; doi:10.1186/s12985-021-01559-3)

## Slide 1
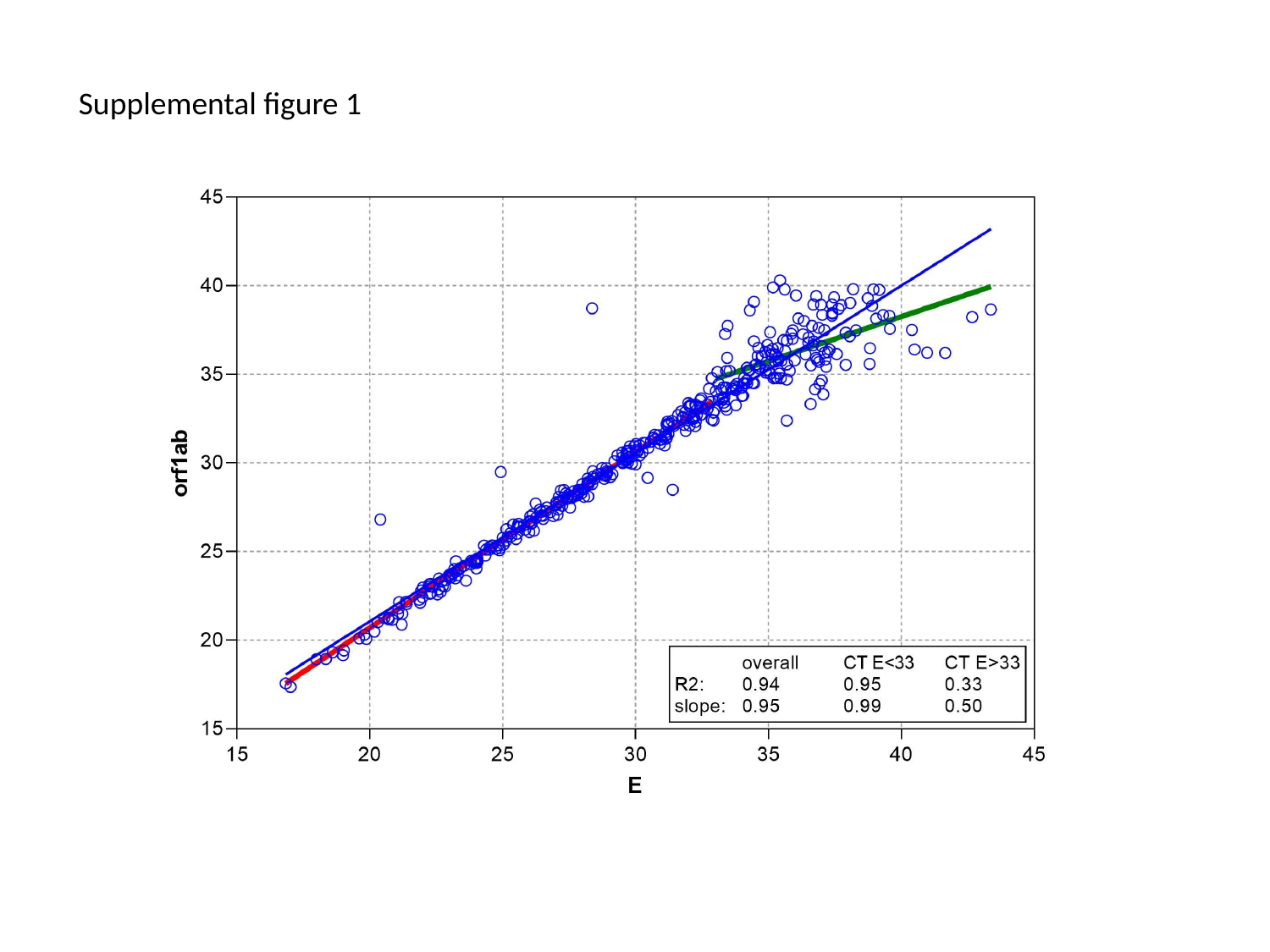

Supplemental figure 1

Supplement: Supplementary file 1 — Additional file 1: Figure S1. Correlation of the CT values obtained for 407 specimens positive for the E-Gene PCR with the orf1ab PCR. Shown is the correlation over all specimens (blue line, R2 = 0.94), for specimens with E-Gene PCR CT values below CT = 33 (red line, R2 = 0.95) and for specimens with E-Gene PCR CT values above CT = 33 (green line, R2 = 0.33). [file 12985_2021_1559_MOESM1_ESM.pptx]
